# Anesthetic management and complications during transvenous pacemaker implantation in dogs

**DOI:** 10.3389/fvets.2026.1754437

**Published:** 2026-03-10

**Authors:** Gabrielly Moreira dos Santos de Oliveira, Mayara Travalini de Lima, Alessandro Rodrigues de Carvalho Martins, Adan William de Melo Navarro, Daniele Midori Kakimoto Higa, Renata Sayuri Akabane, Denise Tabacchi Fantoni

**Affiliations:** 1 Department of Surgery and Anesthesiology of Faculty of Veterinary Medicine, Unit of Training Applied to Research and Extension (UFAPE), Veterinary Intensive Care Unit, São Paulo, Brazil; 2 Independent Practitioner, São Paulo, Brazil; 3 Department of Surgery, Faculty of Veterinary Medicine and Animal Science, University of São Paulo, São Paulo, Brazil

**Keywords:** anesthesia, atrioventricular block, dog, hemodynamics, pacemaker

## Abstract

**Introduction:**

With the growing number of veterinary centers performing pacemaker implantation in small animals, a better understanding of anesthetic management of these procedures has become essential. Most available studies focus primarily on surgical techniques, with limited discussion of anesthetic safety, efficacy, and intraoperative complications.

**Materials and methods:**

Eighteen anesthetic procedures for transvenous pacemaker implantation were retrospectively reviewed in dogs treated at a veterinary referral center in São Paulo, Brazil, between 2024 and 2025. All dogs underwent clinical and laboratory evaluation, electrocardiography and/or Holter monitoring, and echocardiography. Continuous intraoperative monitoring included heart rate, electrocardiography, respiratory rate, end-tidal CO_2_, oxygen saturation, temperature, and invasive or non-invasive arterial blood pressure. Collected data included age, breed, sex, anesthetic protocol, clinical conditions, intraoperative events, and outcomes. Continuous variables were expressed as mean ± SD or median (range) according to data distribution assessed by the Shapiro–Wilk test. Group comparisons were performed using Student's t-test or Mann–Whitney U test, and categorical variables were analyzed using chi-square or Fisher's exact test (*p* < 0.05).

**Results:**

Sixteen dogs (8 males and 8 females) underwent 18 anesthetic procedures, including two reinterventions. The main indication for pacemaker implantation was third-degree atrioventricular block (75%). Anesthetic protocols were individualized according to patient comorbidities. The most frequent anesthetic complication was hypotension (27.7%), successfully managed with fluid therapy and vasoactive support. Electrode migration occurred in 11% of procedures and required reintervention. No perioperative deaths were recorded.

**Discussion and conclusion:**

Transvenous pacemaker implantation in dogs was successfully performed under individualized anesthetic management despite heterogeneous clinical conditions and absence of a standardized protocol. Hypotension was the most common anesthetic complication but was effectively treated. The routine use of temporary external pacing provided an essential safety margin for maintaining perioperative hemodynamic stability. No perioperative deaths were recorded. These findings support the feasibility and safety of tailored anesthetic strategies for canine pacemaker implantation, although prospective studies with larger populations are warranted to define optimal standardized protocols.

## 1 Introduction

Since the first report on pacemaker implantation in a dog in 1967, there has been substantial progress, with improved patient prognosis and increased treatment adherence in veterinary medicine (1). The use of transvenous implants, inserted via minimally invasive surgery, allows placement of an electrode into the right ventricle via jugular catheterization; in dual-chamber implants, an additional electrode is positioned in the right atrium. The generator is then implanted in the dorsocervical region or “neck,” but also in other locations (such as in the area of the lateral thorax, typically in the craniodorsal aspect of the scapula) within the subcutaneous tissue for permanent fixation and choice depends of the patient size/body conformation, skin status, expected level of activity of the patient and veterinary cardiologist's preference (2).

The most common causes of bradyarrhythmias requiring pacemaker implantation are advanced second-degree atrioventricular block (AVB), frequently associated with second-degree Mobitz type II AVB, third-degree or complete atrioventricular block (third-degree AVB or CAVB), vasovagal syncope, and persistent atrial standstill in symptomatic patients (3). Certain diseases may predispose animals to bradyarrhythmias, such as hypothyroidism, in which arrhythmias result from reduced oxygen delivery and consumption due to decreased cardiac output and altered adrenergic sensitivity, or from direct effects of thyroid hormones on the heart. Consequently, bradyarrhythmia may occur in 5–26% of hypothyroid patients, with first-degree AVB being the most common. However, second-degree AVB and atrial fibrillation may also occur, as well as dilated cardiomyopathy with cardiac remodeling and sick sinus syndrome (4, 5).

Several breeds, namely Labrador Retriever, West Highland White Terrier, Springer Spaniel, Boxer, mixed-breed short-haired dogs, Cavalier King Charles Spaniel, Dachshund, and Standard Schnauzer, may be at risk of severe bradyarrhythmia (6–8). Considering this, a comprehensive preoperative assessment may be indicated, with electrocardiography and periodic cardiac monitoring recommended for these patients.

Transvenous pacemaker implantation is a delicate procedure that has undergone major technical improvements. However, several complications have been reported, including electrode displacement, pacemaker malfunction due to generator disconnection, inadequate stimulation caused by electrode fracture, infection at the generator site, premature battery depletion, pulmonary thromboembolism, intraoperative arrhythmias such as ventricular tachycardia or fibrillation, and risk of cardiac perforation (6, 8, 9).

The anesthetic plan for this procedure should be individually tailored, with particular emphasis on premedication and peri-anesthetic drug selection, to preserve cardiovascular function and cardiac output while minimizing vasodilation and hypotension associated with low-output states. Anaesthetic agents and doses should be selected based on the patient's clinical history and comorbidities, including structural cardiac abnormalities identified on echocardiography, as well as the dog's current clinical presentation (8, 10).

Reported anaesthetic regimens vary and include premedication with butorphanol and atropine, acepromazine and morphine, butorphanol alone, butorphanol and glycopyrrolate, meperidine, methadone, morphine, and buprenorphine. When the dog is cooperative, administration of sedatives or tranquilizers may not be required. Induction agents reported include midazolam combined with etomidate, propofol, alfaxalone, etomidate with lidocaine, ketamine with diazepam, and occasionally thiopental. Anesthesia maintenance was performed with isoflurane, sevoflurane, or halothane (7–11).

The objective of this study was to describe the peri-anesthetic management of pacemaker implantation procedures at our institution and to review morbidity and mortality and clinical decision-making.

## 2 Materials and methods

### 2.1 Ethical approval

This retrospective study evaluated perioperative data from dogs that underwent transvenous pacemaker implantation between 2024 and 2025 at the UFAPE Veterinary Intensive Care Unit, São Paulo, Brazil. The study was based exclusively on pre-existing clinical data obtained from medical records and, in accordance with institutional policy and national ethical guidelines (CONCEA), COPE Council (2019), OLAW/IACUC (2002), American Veterinary Medical Association Guidelines (AVMA 2020), was classified as exempt from submission to the Institutional Ethics Committee for the Use of Animals (CEUA), as no direct intervention on the animals was performed. Written informed consent from owners was not required because this was a retrospective case series based on previously collected medical data rather than prospectively obtained information. All animals were treated in accordance with the institutional standard of care.

### 2.2 Study design and case selection

This was a single-center, retrospective, pragmatic cohort study. Data were obtained from 18 anesthesia records and medical charts describing pacemaker implantation procedures performed from March 2024 to March 2025. Recorded variables included breed, age, weight, sex, preexisting diagnosis, and cardiac abnormalities identified by echocardiography, Holter, or electrocardiography, as well as the American Society of Anesthesiologists (ASA) physical status classification.

Two dogs required reintervention and were therefore counted twice. All dogs were symptomatic for bradyarrhythmia and deemed eligible for transvenous pacemaker implantation by the Cardiology Service of the UFAPE Veterinary Hospital. Inclusion criteria comprised dogs that had undergone thorough physical examination, complete clinical evaluation, and detailed owner history, as well as laboratory testing (hematology and serum biochemistry). Biochemical analyses included hepatic parameters [alanine aminotransferase (ALT), alkaline phosphatase (ALP), and gamma-glutamyl transferase (GGT)] and renal parameters (urea and creatinine). Electrocardiography and/or Holter monitoring and echocardiography were performed to evaluate cardiac rhythm and morphology in correlation with the clinical history in all patients.

Among the included dogs, one was pregnant (39–43 days of gestation) and underwent pacemaker implantation uneventfully; fetal viability was confirmed postoperatively, and the bitch later delivered five puppies naturally, with one stillbirth attributed to fetal distress. One dog had diabetes mellitus and idiopathic epilepsy requiring perioperative insulin infusion, and one presented with right-sided congestive heart failure at the time of implantation.

### 2.3 Anesthetic management and monitoring

Before anesthetic induction, all dogs were pre-oxygenated with 100% oxygen for 5 min. During this period, continuous electrocardiographic (ECG) monitoring was initiated, and animals were prepared for external cardiac pacing. Adhesive pacing pads were placed bilaterally over the thorax at the point of maximal impulse corresponding to the mitral valve area (fifth intercostal space). The external pacemaker (Umed 20, Mindray Animal Medical Technology Co) was set to demand mode, with the minimum heart rate and current intensity individually adjusted for each dog (Supplementary materials 1–3).

Premedication was avoided whenever possible due to the presence of clinically significant bradyarrhythmias. When mild sedation was required, butorphanol was administered. In one highly anxious dog, acepromazine was added.

Anesthetic induction protocols varied according to each patient's clinical condition and included combinations of ketamine, midazolam, etomidate, and propofol. Maintenance of anesthesia was achieved using total intravenous anesthesia with ketamine–midazolam or propofol–midazolam infusions, inhalational isoflurane, or balanced combinations, according to the individual patient status. Detailed drug combinations and infusion rates for premedication, induction, and maintenance are provided in Table 1. In dogs with right-sided congestive heart failure and in the late-gestation pregnant dog, ketamine was avoided.

**Table 1:** Anesthetic protocols used in 16 dogs undergoing transvenous pacemaker implantation at the Faculty of Veterinary Medicine, Unit of Training Applied to Research and Extension (UFAPE), Veterinary Intensive Care Unit, São Paulo, Brazil.

**Anesthetic protocols**	**Patients**
**Pre-anesthetic medication**	**Number of patients**
Butorphanol (0.1–0.3 mg.kg^−1^, IM)	9
Acepromazine (0.03 mg.kg^−1^, IM)	1
No premedication	8
Total	18^*^
**Induction**	**Number of patients**
Ketamine (5 mg.kg^−1^, IV) and Midazolam (0.5 mg.kg^−1^, IV)	6
Propofol (2.5 mg.kg^−1^, IV) and Midazolam (0.5 mg.kg^−1^, IV)	1
Patient induced in ICU	1
Etomidate (1-2 mg.kg^−1^, IV) and Midazolam (0.3-0.5 mg.kg^−1^, IV)	2
Propofol (4 mg.kg^−1^, IV) and ketamine (1 mg.kg^−1^, IV)	1
Ketamine (1-5 mg.kg^−1^, IV) and Midazolam (0.3-0.5 mg.kg^−1^, IV) and Propofol (1-5 mg.kg^−1^, IV)/Etomidate (0.5-2 mg.kg^−1^, IV)	7
Total	18^*^
**Maintenance**	**Number of patients**
Ketamine (0.6-1.8 mg.kg.h^−1^, IV) and Midazolam (0.3-0.5 mg.kg.h^−1^, IV) and Isoflurane	10
Ketamine (0.6 mg.kg.h^−1^, IV) and Midazolam (0.5 mg.kg.h^−1^, IV)	1
Isoflurane and Fentanyl (10 mcg.kg.h^−1^, IV)	1
Isoflurane	1
Isoflurane and Propofol (0.1 mg.kg.min^−1^, IV) and Rocuronium (0.6 mg.kg.h^−1^, IV)	2
Isoflurane and Midazolam (0.3 mg.kg.h^−1^, IV)	1
Propofol (0.4 mg.kg.min^−1^, IV) and Midazolam (0.2 mg.kg.h^−1^, IV)	1
Ketamine (0.6-1.2 mg.kg.h^−1^, IV) and Midazolam (0.3-0.5 mg.kg.h^−1^, IV) and Isoflurane and Rocuronium (0.6 mg.kg.h^−1^, IV)	1
Total	18^*^

^*^Anesthetic protocols used in two re-intervention procedures were also included.

Following anesthetic induction and endotracheal intubation, dogs were connected to a rebreathing anesthesia circuit (Dräger Fabius; Drägerwerk AG & Co., Lübeck, Germany). Immediately after induction, the external pacemaker was switched from demand mode to fixed mode to maintain a minimum heart rate between 30 and 50 beats per minute (bpm). Further adjustments to pacing parameters were made intraoperatively as necessary based on continuous ECG monitoring and hemodynamic status.

Mechanical ventilation was instituted using either pressure-controlled or volume-controlled modes, selected according to patient synchrony and the presence of comorbidities. Ventilatory settings were adjusted to maintain normocapnia, defined as an end-tidal carbon dioxide concentration (EtCO_2_) between 35 and 45 mmHg. Tidal volume was typically set at 10–15 ml/kg, inspiratory pressure at approximately 10 cmH_2_O, and positive end-expiratory pressure (PEEP) at 5 cmH_2_O. When required to facilitate ventilatory management, neuromuscular blockade was achieved with rocuronium administered intravenously at a dose of 0.6 mg·kg^−1^ IV followed by continuous infusion (0.3–0.6 mg·kg^−1^·h^−1^ IV) if prolonged blockade was necessary. Neuromuscular blockade was assessed using a TOF Guard monitor through train-of-four (TOF) peripheral nerve stimulation. Four supramaximal electrical stimuli at a frequency of 2 Hz were delivered, and the depth of neuromuscular blockade was evaluated based on the number and fade of evoked muscular responses, as well as the train-of-four ratio.

Throughout anesthesia, all dogs were continuously monitored using a multiparameter monitor (Dräger Vista 120, Drägerwerk AG & Co. KGaA, Lübeck, Germany). Recorded variables included heart rate (HR), ECG for rhythm analysis, respiratory rate (RR), capnography, ETCO_2_, peripheral oxygen saturation (SpO_2_), body temperature, and arterial blood pressure. Invasive arterial blood pressure was preferentially measured using a disposable pressure transducer (TruWave, Edwards Critical Care Division, USA), allowing continuous recording of systolic (SAP), diastolic (DAP), and mean arterial pressure (MAP). When invasive arterial catheterization was not feasible, systolic arterial pressure was measured using Doppler ultrasound (Parks Medical Electronics^®^, Model 811-B, Aloha, OR, USA).

Anesthetic management aimed to maintain arterial blood pressure within the normotensive range in order to preserve the physiological renal autoregulatory zone and ensure overall hemodynamic stability. According to the American College of Veterinary Internal Medicine (ACVIM) guidelines (12), arterial hypertension was defined as systolic arterial pressure (SAP) >160 mmHg, whereas hypotension was defined as a mean arterial pressure (MAP) < 65 mmHg or systolic arterial pressure (SAP) < 90 mmHg (13). Body temperature was actively monitored and managed throughout anesthesia using warming devices and environmental adjustments as required.

Intraoperative hypotension was managed according to a predefined treatment protocol. Initial management consisted of reducing anesthetic depth and administering a crystalloid fluid bolus using Lactated Ringer's solution (10 ml·kg^−1^ over 15 min). When hypotension persisted despite these measures, vasoactive drug support was instituted. Continuous intravenous infusions of norepinephrine (0.1–0.8 μg·kg·min^−1^) and/or dobutamine (1–7 μg·kg·min^−1^) were administered, with dose adjustments based on continuous hemodynamic monitoring and clinical response.

Anesthetic complications were predefined as clinically relevant physiological disturbances occurring during the anesthetic period and were classified according to objective monitoring thresholds. Cardiovascular complications included tachycardia and bradycardia, defined as heart rate values persistently above or below species- and size-adjusted reference ranges, as well as arterial hypotension and hypertension according to the criteria described above. Cardiac arrhythmias were defined as any abnormal rhythm detected by continuous ECG monitoring. Thermoregulatory complications included hypothermia, defined as core body temperature <37.5 °C, and hyperthermia, defined as core body temperature >39.5 °C. In addition, intraoperative technical complications with potential impact on anesthetic management or patient stability were recorded.

Intraoperative arterial hypertension was managed according to a predefined treatment protocol based on the presence or absence of surgical stimulation. When hypertension occurred without concurrent surgical manipulation, sodium nitroprusside was administered as a continuous intravenous infusion (1 ug.kg.min^−1^). When hypertension occurred during surgical stimulation, analgesic supplementation was provided using continuous infusions of fentanyl (2–10 μg·kg^−1^·h^−1^, IV) or remifentanil (6 μg·kg^−1^·h^−1^, IV).

Postoperative analgesia was provided with metamizole (25 mg·kg^−1^, IV) and anti-inflammatory therapy (meloxicam 0.1 mg·kg^−1^, IV or dexamethasone 0.1 mg·kg^−1^, IV). Additional opioids were administered according to clinical response. Rescue analgesia consisted of methadone (0.1 mg·kg^−1^, IV), morphine (0.1–0.3 mg·kg^−1^, IV), butorphanol (0.1–0.2 mg·kg^−1^, IV), or tramadol (4 mg·kg^−1^, IM). Formal pain scoring was not applied retrospectively; analgesic supplementation was guided by anticipated surgical nociceptive intensity and intraoperative autonomic responses.

In diabetic patients, perioperative glucose control was achieved through continuous insulin infusion and glucose supplementation, guided by serial blood glucose measurements performed every 30 min, following established clinical recommendations (14). Regular insulin was diluted in a 250 ml bag of 0.9% sodium chloride or Plasma-Lyte A and administered intravenously, with infusion rates adjusted according to blood glucose concentration. When blood glucose exceeded 250 mg·dl^−1^, insulin (2.2 IU·kg^−1^) diluted in a 250 ml bag of 0.9% sodium chloride or Plasma-Lyte A was infused at 10 ml·h^−1^. As blood glucose decreased to 200–250 mg·dl^−1^, intravenous fluids were supplemented with 2.5% dextrose, and the insulin infusion rate was reduced to 7 ml·h^−1^. For blood glucose concentrations between 100–200 mg·dl^−1^, Plasma-Lyte A supplemented with 2.5% dextrose was maintained, with insulin infusion at 5 ml·h^−1^. When blood glucose fell below 100 mg·dL^−1^, fluids containing 5% dextrose were administered, and insulin infusion was discontinued.

Pacemaker programming followed standardized nomenclature and recommendations from the Heart Rhythm Society (HRS) and the British Pacing and Electrophysiology Group (BPEG). Devices were programmed as single- or dual-chamber systems according to individual clinical requirements. Single-chamber pacemakers were set in VVI or VVIR modes, providing ventricular sensing and pacing with inhibition of stimulation in the presence of intrinsic ventricular activity, with optional rate-responsive function. Dual-chamber devices were programmed in VDD or DDD modes, enabling atrial sensing with ventricular pacing or full atrioventricular sensing and pacing to preserve atrioventricular synchrony. Minimum pacing rates were individually adjusted according to hemodynamic status. A descriptive table summarizing pacemaker mode nomenclature and functional characteristics is provided to facilitate interpretation of programming modes (Table 2).

**Table 2:** The Heart Rhythm Society (HRS) and British Pacing and Electrophysiology Group (BPEG) guide to pacemaker modes of operation.

**I**	**II**	**III**	**IV**	**V**
Chamber (s) Paced	Chamber (s) Sensed	Mode (s) of Response	Programmable Functions	Antitachycardia Functions
V: Ventricle	V: Ventricle	T: Triggered	R: Rate modulated	O: None
A: Atrium	A: Atrium	I: Inhibited	C: Communicating	P: Paced
D: Dual (A and V)	D: Dual (A and V)	D: Dual (T and I)	M: Multi programmable	S: Shocks
O: None (S: Single)	O: None (S: Single)	O: None	P: Simple programmable O: None	D: Dual (P and S)

Adapted from DeForge et al., (1).

For each case, the analyzed variables included breed, age, sex, anesthetic protocol, clinical conditions potentially affecting perioperative safety, observed complications, administered treatments, and the type of pacemaker implant.

## 3 Statistical analysis

Continuous variables were expressed as mean ± standard deviation for normally distributed data and as median and range for non-normally distributed data. Data distribution was assessed using the Shapiro–Wilk test and visual inspection of histograms. Normally distributed continuous variables were compared between groups using the Student's *t*-test, whereas non-normally distributed data were analyzed using the Mann–Whitney *U* test.

Categorical variables were presented as absolute numbers and relative frequencies (percentages) and were analyzed using the chi-square test or Fisher's exact test, as appropriate. A significance level of *P* < 0.05 was adopted for all statistical analyses. Statistical analyses were performed using IBM SPSS Statistics for Windows, version 25 (IBM Corp., Armonk, N.Y., USA).

## 4 Results

A total of 18 transvenous pacemaker implantation procedures were performed, including two reinterventions due to electrode migration. Therefore, 16 dogs underwent anesthesia. Eight were males (50%) and eight females (50%). Median age was 6 years (range 1–12), and median body weight was 8.9 kg (range 3.1–40). Mixed-Breed dogs were the most frequent group (three cases; Table 3).

**Table 3:** Demographic data of 16 dogs included in the study, which underwent transvenous pacemaker implantation at the Faculty of Veterinary Medicine, Unit of Training Applied to Research and Extension (UFAPE), Veterinary Intensive Care Unit, São Paulo, Brazil.

**Breed**	**Number**	**Sex**	**Age (years)**	**Weight (kg)**	**Underlying condition**
German spitz	1	Male	4	7	Atrial standstill
	1	Male	6	3	Sick sinus syndrome
Shih-tzu	1	Male	11	8	Third-degree AV block
	1	Female	6	6	Third-degree AV block
Maltese	1	Female	2	8	Third-degree AV block
Pit bull	1	Male	3	30	Third-degree AV block
English cocker spaniel	1	Female	10	17	Second-degree AV block (Mobitz type II)
Akita	1	Male	8	40	Third-degree AV block
Chow-chow	1	Male	10	19	Third-degree AV block
Cane Corso	1	Male	1	37	Third-degree AV block
Mixed-breed	1	Female	1	8	Third-degree AV block
	1	Female	4	4	Third-degree AV
	1	Female	11	11	Third-degree AV block
West highland white terrier	1	Male	12	8	Sick sinus syndrome
White swiss shepherd	1	Female	10	31	Third-degree AV block
Pug	1	Female	3	7	Third-degree AV block
Total	16				

Median physiological parameters were heart rate 60 bpm (range 21–204), respiratory rate 18 breaths/min (7–55), end-tidal CO_2_ 36 mmHg (20-47), SpO_2_ 97% (80–100), end-tidal isoflurane 1.0% (0–2.0), and body temperature 37.8 °C (34.5–38.9). Mean arterial pressures were: SAP 122 ± 28 mmHg, MAP 83 ± 17 mmHg, and DAP 64 ± 20 mmHg.

The main indication for pacemaker implantation was third-degree atrioventricular block in 12 dogs (75%), followed by sick sinus syndrome in two (12.5%), second-degree Mobitz type II AV block in one (6.25%), and atrial standstill in one (6.25%). Echocardiographic abnormalities were present in most dogs, primarily mitral valve insufficiency (eight cases). One dog had regurgitation of all four cardiac valves and right-sided congestive heart failure with ascites requiring preoperative abdominocentesis.

Premedication was administered in 10 anesthetic events: butorphanol in nine and acepromazine–butorphanol in one. Eight anesthetic events were performed without premedication. Induction protocols varied: ketamine–midazolam in six, etomidate–midazolam in two, propofol–midazolam in one, propofol–ketamine in one, and mixed combinations of ketamine, midazolam, propofol, or etomidate in seven. One patient was sedated in the intensive care unit before transfer to the operating room.

Anesthetic maintenance most consisted of ketamine–midazolam infusions combined with isoflurane (10 events). Other techniques included ketamine–midazolam alone (one), isoflurane alone (one), isoflurane–fentanyl (one), isoflurane–propofol–rocuronium (two), isoflurane–midazolam (one), propofol–midazolam (one), and ketamine–midazolam–isoflurane–rocuronium (one).

One epileptic–diabetic dog underwent anesthesia under continuous propofol and midazolam infusion and required intraoperative glycemic control with insulin and glucose infusions.

Mean anesthetic duration was 98 min (range 60–150).

Intraoperative systemic hypertension (SAP >160 mmHg) occurred in 10 anesthetic events (55.5%). Three hypertensive episodes occurred in patients receiving continuous vasoactive infusions, including one requiring sodium nitroprusside in addition to discontinuation of vasoactive support. One episode occurred during resuscitation following cardiopulmonary arrest with epinephrine administration. Three hypertensive episodes were temporally associated with surgical stimulation and were treated with opioid rescue (fentanyl, remifentanil, or morphine). Two dogs developed a single transient hypertensive peak that resolved without intervention. One dog developed hypertension in the immediate postoperative period despite analgesic rescue and received acepromazine (0.02 mg·kg^−1^, IV) for agitation-associated hypertension.

Postoperative opioid supplementation was administered during the final phase of anesthesia before emergence, while patients were still unconscious, in seven cases: morphine in four, methadone in two, and tramadol in one.

Hypotension (MAP < 65 mmHg) occurred in five anesthetics events (27.7%) and was promptly treated with crystalloid boluses and, when required, norepinephrine and/or dobutamine infusions. No persistent adverse clinical consequences were observed. One patient received only norepinephrine, one received only dobutamine and three received both.

One pacemaker-dependent dog developed cardiopulmonary arrest during generator replacement; return of spontaneous circulation was achieved after prompt resuscitation. Two dogs required reintervention due to electrode migration, one immediately and one 24 h postoperatively; both were counted as separate anesthetic events.

One dog developed intraoperative hyperthermia (39.6 °C), which resolved with active cooling.

Pacemaker programming included 12 single-chamber devices (VVI/VVIR) and four dual-chamber devices (VDD/DDD). Minimum pacing rates ranged from 50 to 90 bpm.

All dogs recovered and were monitored in the intensive care unit for approximately 24 h. No deaths occurred during the immediate postoperative period.

## 5 Discussion

Although previous studies have reported breed predispositions for pacemaker implantation, no consistent pattern was observed in the present study, likely due to the small sample size. Mixed-breed dogs were the most common (3/16), followed by isolated cases of Labrador Retriever, American Cocker Spaniel, Dachshund, Miniature Schnauzer, and West Highland White Terrier, partially aligning with previous reports (2, 6–9). The main indication for pacemaker implantation was associated with third-degree atrioventricular block (AVB 3, 75%), followed by second-degree AV block Mobitz type II, sick sinus syndrome, and atrial standstill. These indications are consistent with previous studies by Noszczyk-Nowak et al. (6), Johnson et al. (7), and Sanchis-Mora et al. (8), all identifying AVB 3° as the predominant diagnosis.

Anesthetic protocols were individualized according to each patient's clinical condition. Whenever possible, the base protocol consisted of butorphanol as premedication—or none when avoided—and induction with ketamine and midazolam. This choice was guided by the cardiovascular stability and sympathomimetic effects of ketamine combined with the minimal hemodynamic impact of midazolam. However, ketamine was avoided in dogs with cardiac insufficiency due to its potential negative inotropic effect at higher doses. Few studies specifically address anesthetic management during pacemaker implantation, as most publications focus on surgical techniques. Nevertheless, the use of opioid premedication followed by dissociative anesthesia (ketamine with diazepam or midazolam), or etomidate–midazolam induction with inhalant maintenance, has been described in dogs (8, 10). In human medicine, pacemaker implantation may be performed under sedation or general anesthesia, depending on patient stability, with induction commonly using propofol and remifentanil (15). Both fentanyl and dexmedetomidine must be used cautiously due to their potential to induce bradycardia (16). Butorphanol was selected in 50% of cases for its sedative properties and cardiorespiratory stability (17, 18). Ketamine–midazolam was the most frequent induction combination, as ketamine provides sympathetic stimulation supporting arterial pressure and cardiac output, while midazolam provides hypnosis and muscle relaxation with minimal cardiovascular depression (19, 20).

For anesthetic maintenance, inhalant anesthetics were generally avoided until pacemaker implantation due to their dose-dependent vasodilatory effects, which could exacerbate hypotension in patients with severe bradyarrhythmia (21). Instead, ketamine–midazolam continuous infusions were preferred to maintain stable mean arterial pressure and support heart rate through sympathetic stimulation. After electrode placement, anesthesia was transitioned to isoflurane. Although isoflurane and sevoflurane have minimal direct myocardial depressive effects compared with halothane, their vasodilatory action remains clinically relevant, particularly in patients predisposed to low cardiac output (22). Therefore, the chosen approach aimed to minimize vasodilation before definitive pacing while maintaining hemodynamic stability.

Special clinical conditions required further protocol adjustments. In the dog with right-sided congestive heart failure, pre-oxygenation with 100% oxygen was used to reduce alveolar hypoxia and the risk of hypoxic pulmonary vasoconstriction, which can increase pulmonary vascular resistance and worsen right ventricular function (23, 24). Ketamine was avoided to prevent further increases in pulmonary vascular resistance and right ventricular afterload (19, 25). Mechanical ventilation with low PEEP was selected to balance oxygenation and venous return.

In the pregnant dog (39–43 days of gestation), induction was achieved with propofol and midazolam. Midazolam was used to reduce propofol requirements and was antagonized with flumazenil at recovery. Propofol alone was avoided to minimize maternal hypotension and myocardial depression (20, 26). Available evidence suggests benzodiazepines are not conclusively associated with congenital malformations in humans or dogs (27–30). Species differences in placentation further suggest reduced fetal drug transfer in dogs (31). Rocuronium was selected for neuromuscular blockade due to its limited placental transfer and documented safety in human obstetric anesthesia (32).

In the epileptic–diabetic patient, total intravenous anesthesia with propofol and midazolam was selected. Ketamine was avoided due to concerns regarding cortical excitability and potential increases in intracranial pressure in neurologic patients, particularly during continuous infusion or jugular manipulation during pacemaker placement (19, 33). Propofol was preferred for its neuroprotective properties and ability to reduce cerebral metabolic demand and intracranial pressure, while midazolam provided anticonvulsant effects and hemodynamic stability (20, 34). Insulin and glucose infusions were titrated every 30 min to maintain glycemic control.

Hemodynamic complications represented the most relevant anesthetic challenges. Hypotension occurred in 27.7% of cases and was successfully managed with volume expansion and vasoactive infusions. No clear correlation was identified between anesthetic protocol and timing of hypotension. anesthesia, particularly in patients with underlying cardiac disease, and has been consistently reported in clinical studies, including dogs undergoing interventional cardiology procedures such as transvascular patent ductus arteriosus occlusion and balloon valvuloplasty, with higher incidences described in other invasive transvascular cardiology interventions performed under inhalant anesthesia, where rates ranging from 63% to 79% have been reported (7–9, 35, 36). In patients who did not respond adequately to norepinephrine, dobutamine was added as a therapeutic trial. At the time of intervention, the precise mechanism of hypotension could not be definitively determined; however, failure to respond to vasopressor therapy raised suspicion of a low-cardiac-output component rather than isolated vasodilation. In this context, adding an inotropic agent represented a physiologically guided strategy to enhance myocardial contractility and forward flow when vasoconstrictor support alone proved insufficient. Dobutamine is a β1-adrenergic agonist that increases intracellular cAMP and improves stroke volume and cardiac output with relatively limited chronotropic effect. This pharmacologic approach is consistent with cardiovascular physiology and clinical literature describing dobutamine as an appropriate inotropic option when hypotension persists despite vasopressor therapy or when impaired cardiac output is suspected (37, 38).

While hypotension represented the main anesthetic challenge in terms of maintaining tissue perfusion, systemic hypertension was equally frequent and multifactorial in origin. Three hypertensive events occurred in dogs receiving continuous vasoactive infusions, including one requiring sodium nitroprusside in addition to discontinuation of vasopressor support, suggesting that excessive vascular tone secondary to catecholamine administration contributed to these episodes (39). Another hypertensive event occurred during cardiopulmonary resuscitation following cardiac arrest, in which epinephrine had been administered, a response consistent with the potent α-adrenergic vasoconstrictive effects of exogenous catecholamines as described in standard anesthetic physiology texts (40).

In contrast, three hypertensive episodes were temporally associated with surgical stimulation and resolved after opioid rescue, supporting a nociception-mediated sympathetic response. This interpretation is consistent with clinical evidence demonstrating that nociceptive surgical stimuli elicit sympathetic activation capable of increasing arterial pressure during general anesthesia, and that opioid administration attenuates these responses by suppressing nociceptive transmission and associated sympathetic outflow (41). Two additional dogs exhibited brief isolated hypertensive peaks that resolved spontaneously without intervention. Such transient hemodynamic fluctuations may reflect autonomic variability during general anesthesia, as anesthetic agents modulate sympathetic–parasympathetic balance and cardiovascular control even in the absence of overt nociceptive stimulation. Changes in heart rate variability and hemodynamic parameters have been demonstrated during general anesthesia, underscoring the dynamic modulation of autonomic cardiovascular control in anesthetized patients (42). Finally, one dog developed hypertension in the immediate postoperative period despite analgesic supplementation and required acepromazine for agitation-associated sympathetic activation. Emergence delirium during recovery from general anesthesia is a recognized phenomenon in both humans and dogs and has been associated with transient sympathetic activation and cardiovascular instability in the early postoperative period. Therefore, agitation-associated autonomic activation represents a plausible mechanism for the postoperative hypertensive episode observed in this study (43, 44).

Postoperative opioid supplementation was administered during the final phase of anesthesia, before patient awakening from anesthesia, while patients were still unconscious, in seven patients. Rescue analgesia was guided by the predicted nociceptive intensity of each surgical stage and individual patient temperament rather than by formal pain scoring systems. This approach was adopted because currently validated composite pain scales in small animals rely primarily on behavioral assessment in conscious patients and are not applicable during deep general anesthesia or when neuromuscular blockade or external pacing precludes behavioral evaluation (45). Consequently, intraoperative analgesic planning in veterinary anesthesia commonly relies on anticipated surgical nociception and physiological responses when objective pain scoring is not feasible. This strategy is particularly relevant in cardiovascularly compromised patients, in whom autonomic responses may be blunted or confounded by anesthetic depth and concurrent drug effects (40).

Technical pacemaker-related complications were also observed. Lead displacement occurred in two cases—one immediately and one within 24 h postoperatively—representing a recognized complication of transvenous pacemaker implantation. Similar rates of lead dislodgement requiring surgical revision have been reported in canine series, indicating this remains an inherent limitation of current implantation techniques (7, 8). A previous case from our institution (not included in this dataset) developed pericardial effusion secondary to anticoagulant therapy and incomplete lead epithelization, a complication also described in dogs (46). A transient electrode disconnection resulting in asystole occurred in one pacemaker-dependent patient during generator replacement; prompt cardiac compressions and lead repositioning restored spontaneous circulation. Comparable technical complications are only sporadically reported in veterinary literature. In this context, the routine use of transcutaneous external pacing during anesthesia provides an essential safety margin. Additionally, the immediate availability of pre-prepared continuous catecholamine infusions (adrenaline or dopamine) is recommended to facilitate rapid hemodynamic support in the event of sudden pacing failure or severe bradyarrhythmia (46–48).

The main limitations of this study include the small sample size, the lack of breed uniformity, and the heterogeneity of clinical conditions, which precluded anesthetic standardization. In addition, the retrospective nature of the study limits causal inference and restricts direct comparison between anesthetic protocols.

This retrospective case series represents an initial step toward understanding anesthetic management for transvenous pacemaker implantation in dogs. Although a standardized anesthetic protocol was initially sought, the findings suggest that individualized anesthetic approaches, tailored to each patient's clinical condition and cardiovascular status, were associated with acceptable perioperative outcomes. Despite variability in anesthetic management, all procedures were completed successfully, and no perioperative deaths were recorded. These results reinforce the importance of patient-centered anesthetic planning rather than rigid protocol standardization in this population. Larger, prospective, and controlled studies are warranted to systematically compare anesthetic protocols and define optimal anesthetic strategies for dogs undergoing transvenous pacemaker implantation.

## Data Availability

The original contributions presented in the study are included in the article/Supplementary material. Further inquiries can be directed at the corresponding author(s).
